# Sternoclavicular Septic Arthritis Due to Invasive Pneumococcal Infection After Type A Influenza Virus Infection

**DOI:** 10.7759/cureus.38859

**Published:** 2023-05-10

**Authors:** Fumitaka Yoshimura, Junko Kubosaki, Kotaro Kunitomo, Takahiro Tsuji

**Affiliations:** 1 Department of General Medicine, National Hospital Organization Kumamoto Medical Center, Kumamoto, JPN

**Keywords:** immunocompetent patient, influenza virus type a, streptococcus pneumoniae, invasive pneumococcal infection, sternoclavicular arthritis

## Abstract

A 24-year-old female patient who had a type A influenza virus infection prior to admission visited our hospital complaining of a fever and right sternoclavicular pain. Blood culture was positive for penicillin-sensitive Streptococcus pneumoniae (pneumococcus). Magnetic resonance imaging of the right sternoclavicular joint (SCJ) showed a high signal intensity area on the diffusion-weighted images. Consequently, the patient was diagnosed with septic arthritis due to invasive pneumococcus. When a patient complains of gradually increasing chest pain after an influenza virus infection, SCJ septic arthritis should be considered in the differential diagnosis.

## Introduction

Influenza viral infections are commonly complicated by secondary pneumococcal infections, leading to severe illness and increased mortality. Several mechanisms have been suggested for the association between pneumococcal infections and influenza virus infections, such as virus-induced conditions that promote bacterial growth, epithelial cell damage by influenza viruses, and enhancement of the inflammatory response [1]. The sternoclavicular joint (SCJ) is a rare site of septic arthritis (SA), accounting for approximately 1.0-9.0% of all SA, with a mortality rate of approximately 4% [2]. Sternoclavicular joint septic arthritis (SCJSA) typically presents as SCJ pain. *Staphylococcus aureus* is the most common causative organism, whereas *Streptococcus pneumoniae* is rare [3]. SCJSA can lead to serious complications such as osteomyelitis, chest wall abscesses, and mediastinitis. Therefore, prompt diagnosis and treatment are important. Here, we report a rare case of sternoclavicular SA due to an invasive pneumococcal infection after a type A influenza virus infection.

## Case presentation

**Figure 1:**
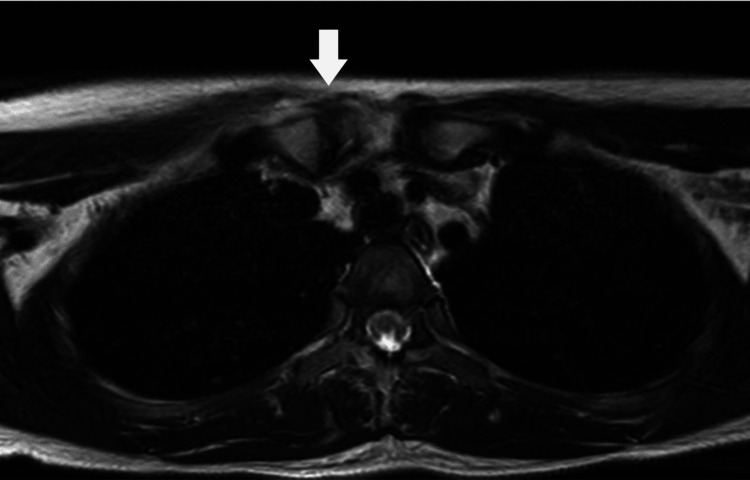
MRI showing a high signal intensity area around the right sternoclavicular joint (arrow) on T2-weighted image.

**Figure 2:**
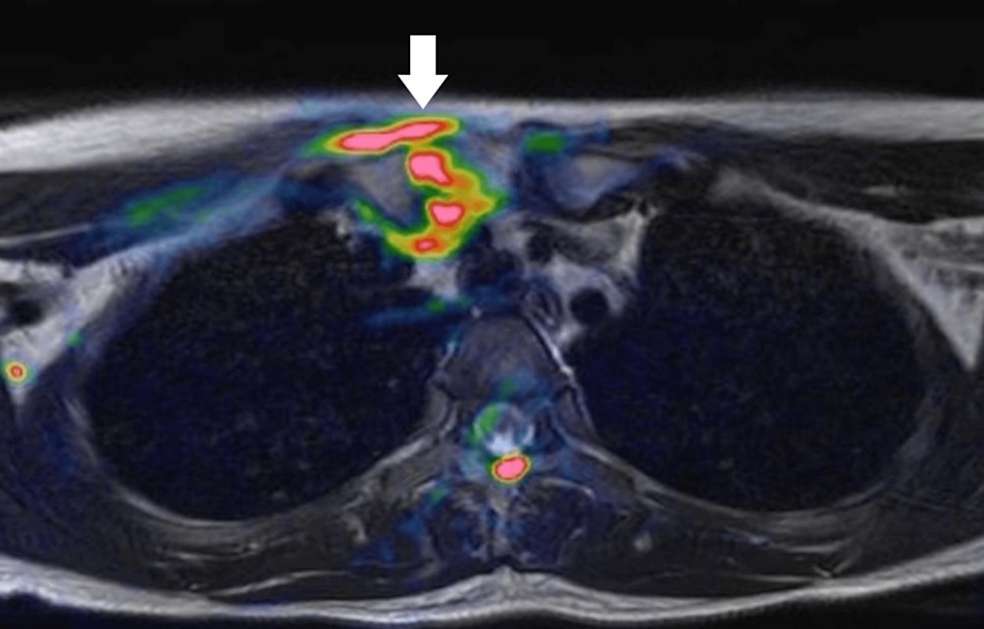
MRI showing a high signal intensity area around the right sternoclavicular joint (arrow) on the diffusion-weighted image (fusion T2-weighted image).

A 24-year-old Asian female patient presented with a five-day history of fever and right sternoclavicular pain. Three weeks before admission, she had a type A influenza virus infection and she took oseltamivir orally (75 mg per dose) twice daily for five days. Physical examination revealed no erythema or swelling of the right SCJ, although pain and tenderness were present. Her neck was slightly stiff. Laboratory tests revealed an elevated C-reactive protein (CRP) level (14.56 mg/dL; normal range < 0.14 mg/dL). There were no autoantibodies against antinuclear or rheumatoid factors present in her serum. Blood culture was positive for penicillin-susceptible *Streptococcus pneumoniae* (pneumococcus). No vegetations were found on a transthoracic echocardiogram. A cerebrospinal fluid (CSF) examination showed no abnormal findings, and *S. pneumoniae* was not detected in the fluid culture. Computed tomography (CT) revealed no significant findings in the SCJ. The patient had an intact spleen and no visible malignancies. Magnetic resonance imaging (MRI) of the right SCJ revealed a high signal intensity area on the T2-weighted image (Figure 1) and on the diffusion-weighted image (Figure 2). SCJ aspiration was not performed because of the low amount of fluid on the MRI. The patient was treated with intravenous antibiotics for four weeks. Ceftriaxone (2 g/day) was used for the first two weeks and sulbactam/ampicillin (9 g/day) for the following two weeks. Her clinical condition and serum inflammatory marker levels improved after the antibiotic therapy. The patient was discharged and was changed to oral sultamicillin (1,125 mg/day) for two weeks at discharge. Thereafter, one month after discharge, the patient's condition was checked and the patient was free of relapse.

## Discussion

*S. pneumoniae* causes bacterial pneumonia, otitis media, sinusitis, and severe invasive infections, especially in children younger than two years and patients older than 65 years [4]. In addition, patients with underlying conditions, such as a splenectomy, diabetes mellitus, and malignancies, are prone to develop these infections [4]. Invasive pneumococcal disease (IPD) commonly presents as bacterial pneumonia, meningitis, or primary bacteremia. However, *S. pneumoniae* can cause infections in any part of the body (such as endocarditis and arthritis), which is known as unusual IPD (uIPD) [5]. Djurdjevic et al. reported a case of IPD and electric purpura in an adult with no underlying disease [6]. The patient was a 67-year-old male with no underlying disease of splenectomy, diabetes, or malignancy. Symptoms were vomiting, diarrhea, and purpura, and it was difficult to suspect invasive pneumococcal infection. It is important to note that invasive pneumococcal infection can occur even in patients without underlying disease. In our case, a 24-year-old female patient had no underlying disease. Her symptoms were fever and SCJ pain. Unlike the case reported by Djurdjevic et al., she was a young patient. We believe that invasive pneumococcal infections cannot be easily ruled out even if the patient is a young adult.

SA is considered a medical emergency because of its high morbidity and mortality rates [4]. *S. aureus* is the most common causative organism of SA in the literature [4]. In contrast, *S. pneumoniae* is an uncommon cause of SA in adults, and a previous study reported that SA developed in only 1.6% of patients with IPD [4]. Pneumococcal arthritis in adults is often reported after middle age, and approximately 80% of patients have risk factors, such as diabetes mellitus, rheumatoid arthritis, malignancy, immunodeficiency, alcohol abuse, or splenectomy [3]. Our patient was a young adult with no known risk factors. The knee is the most frequently infected joint, whereas the SCJ is an uncommon site for pneumococcal arthritis [4].

The SCJ is a rare site of SA, accounting for approximately 1.0-9.0% of all SAs [2]. *S. aureus* is the most common causative pathogen of SCJSA, whereas *S. pneumoniae* is rare [3]. In a review of 180 previously reported SCJSA cases, the mean age of patients was 45 years, and 73% were male [2]. The main risk factors were intravenous drug use (21%), diabetes (13%), and trauma (12%) [2]. However, cases have been reported in healthy adults who developed SCJSA [7,8]. Severe complications such as osteomyelitis (55%), chest wall abscesses (25%), and mediastinitis (13%) were common [2]. And especially in patients with these complications, surgical treatment with resection of the SCJ and ipsilateral pectoralis major muscle flap is preferred [7]. On the other hand, a case of streptococcal SCJSA complicated by chest wall abscess was cured after six weeks of antimicrobial therapy with good sensitivity [9]. With prompt diagnosis and selection of a susceptible antimicrobial agent, the patient may be cured with medical therapy alone.

Influenza virus infections are commonly complicated by secondary pneumococcal infections, leading to severe illness and increased mortality [1]. We could find no reports of pneumococcal SCJ arthritis that developed after influenza virus infection. Several mechanisms have been suggested for the association of *S. pneumoniae* infection with influenza virus infection, such as virus-induced conditions that promote bacterial growth, epithelial cell damage by influenza viruses, and enhancement of the inflammatory response [1,10]. Our patient had no typical symptoms or underlying conditions that could lead to the development of pneumococcal infection. The patient was a young adult. We postulate that the only trigger for her suspected pneumococcal infection was her history of type A influenza infection.

## Conclusions

It is important to identify pneumococcal arthritis following influenza virus infection, even in young adults with healthy immune systems. Complications of SCJSA can be severe diseases such as osteomyelitis, chest wall abscesses, and mediastinitis. When a patient complains of gradually increasing chest pain after an influenza virus infection, this disease should be considered in the differential diagnosis.
